# How a mobile app supports the learning and practice of newly qualified doctors in the UK: an intervention study

**DOI:** 10.1186/s12909-015-0356-8

**Published:** 2015-04-08

**Authors:** Alison Bullock, Rebecca Dimond, Katie Webb, Joseph Lovatt, Wendy Hardyman, Mark Stacey

**Affiliations:** 1 The Cardiff Unit for Research and Evaluation in Medical and Dental Education (CUREMeDE), Cardiff University School of Social Sciences, Glamorgan Building, King Edward VII Avenue, CF10 3WT Wales, UK; 2 Cesagene, Cardiff University, 10/12 Museum Place, CF10 3BG Wales, UK; 3 Capita Project Services, Module 1, Level 2, Friends Life Centre, Bristol, BS34 8SW England UK; 4 Cardiff Business School, Cardiff University, Aberconway Building, Column Drive, Cardiff, CF10 3EU Wales UK; 5 Wales Deanery, School of Postgraduate Medical and Dental Education (PGMDE), Neuadd Meirionnydd, Heath Park, Cardiff University, Cardiff, CF14 4YS Wales UK

**Keywords:** Technology enhanced learning, Workplace learning, Trainee doctors, Smartphones, Transitions, Patient safety

## Abstract

**Background:**

The transition from medical school to the workplace can be demanding, with high expectations placed on newly qualified doctors. The provision of up-to-date and accurate information is essential to support doctors at a time when they are managing increased responsibility for patient care. In August 2012, the Wales Deanery issued the Dr.Companion© software with five key medical textbooks (the iDoc app) to newly qualified doctors (the intervention). The aim of the study was to examine how a smartphone app with key medical texts was used in clinical workplace settings by newly qualified doctors in relation to other information sources and to report changes over time.

**Methods:**

Participants (newly qualified - Foundation Year 1 - doctors) completed a baseline questionnaire before downloading the iDoc app to their own personal smartphone device. At the end of Foundation Year 1 participants (n = 125) completed exit questionnaires one year later. We used Wilcoxon Signed Rank test to analyse matched quantitative data.

**Results:**

We report significant changes in our participants’ use of workplace information resources over the year. Respondents reduced their use of hard-copy and electronic versions of texts on PCs but made more use of senior medical staff. There was no significant difference in the use of peers and other staff as information sources. We found a significant difference in how doctors felt about using a mobile device containing textbooks in front of patients and senior medical staff in the workplace.

**Conclusions:**

Our study indicates that a mobile app enabling timely, internet-free access to key textbooks supports the learning and practice of newly qualified doctors. Although participants changed their use of other resources in the workplace, they continued to consult with seniors. Rather than over-reliance on technology, these findings suggest that the app was used strategically to complement, not replace discussion with members of the medical team. Participants’ uncertainty about using a mobile device with textbook app in front of others eased over time.

## Background

The development of smartphones and tablets with enhanced capacity and function, improved memory, larger screens, the ability to access the internet and download software has resulted in them becoming ever-present within medicine. Through their wide range of uses, including communication, diagnostics, self-monitoring and access to specialist medical software packages or ‘apps’ [1], mobile devices are increasingly employed by medical students and physicians in the workplace [2,3]. Smartphone technology would seem to be part of a technological revolution within medical practice [4]. Identifying and keeping up-to-date with developments in technology to support workplace learning is a key challenge for medical educators. The position and appropriation of technology within the learning sphere is that it should support, serve and develop learning, rather than drive the learning experience [5]. Technology which provides help when needed and is responsive to learners’ developing knowledge and skills can offer a form of dynamic scaffolding [6]. Sfard [7] argues that learners need both to acquire knowledge and participate in learning processes, thereby acknowledging and incorporating the context of learning through participation as well as the individual attainment of knowledge.

The premise that learning entails both knowledge acquisition and participation is central to workplace education and training and is especially relevant at significant points of participatory learning such as during the transition from medical student to newly qualified doctor. The development of learning through the acquisition of explicit knowledge (for example, from textbooks) and processes of learning through participation are both central to the new medical practitioners’ learning experience in the workplace. Mobile technology has the potential to support not only the acquisition of explicit knowledge but also the new doctors’ engagement in the workplace by, for example, supporting their preparation for patient encounters and their dialogue with members of medical teams. However, much of the research on the use of mobile technology is confined to medical curricular and evidence on how mobile resources may support trainee doctors’ learning in the workplace is limited. The pace and spread of developments in mobile technology and medically relevant applications is in stark contrast to the much slower rhythms of research and subsequent publication of evidence.

In this paper we report findings from the evaluation of an intervention which provided newly qualified doctors in Wales with a library of cross-searchable medical texts on their own smartphone devices via an app. The first years of medical practice are a time when rapid access to reliable information resources is essential for learning and practice. While several studies have explored how smartphones can improve communication within education and training, few have considered how smartphones are used as a reference resource within workplace (typically hospital) settings [8].

The main emphasis in current research is on exploring attitudes to smartphone use, estimating the extent and primary purpose of use, and identifying perspectives on potential benefits and challenges [1,9,10]. In terms of availability, there are some differences in the projected numbers of doctors or medical students using mobile technology. One systematic review [11] concluded that within health care, uptake and use of personal digital assistants (PDAs), a forerunner to smartphones, had increased but was variable. More recently, high rates of smartphone ownership amongst medical students and junior doctors have been reported [12]. Another study [13] found high usage, with 77% of medical students in Monash University, Australia, owning a smartphone, of which 76% used medical apps. Students were identified as having positive opinions about smartphones, with the conclusion that smartphone devices have the potential to play a significant role within medical education. A review of the literature on use of PDAs by health professionals and medical students also reported a positive attitude towards their use in medicine [14] and another review found evidence of clinicians making effective use of handheld devices to access information and guidelines and improve diagnostic decisions [15].

The repeated message from research is widespread support for the use of smartphones within medicine. However, the availability of mobile technology does not equate to it being used to enhance learning and training. Concerns have been expressed about the potential of mobile devices to cause doctor distraction [16] and dependency on technology and its use as a substitute for critical clinical thinking [12,17]. The widespread informal use of mobile technology in medical education and the difficulties of researching formal use make it hard to assess benefits to learning and training. Practical barriers to smartphone use have also been recognised including: cost, availability of technology, effective monitoring of use and problems of synchronisation with alternative resources [12].

Although we know a lot about medical students’ and doctors’ views on use of mobile devices in the support of learning, less is known about how they are actually used by newly qualified doctors in practice. In a previous phase of our project, we documented that having access to a smartphone library of medical texts improved user confidence and enhanced patient care [18]. In this article we contribute to the field by examining how smartphones are used in relation to other types of resources available in the workplace and report changes in their use over time. We also consider the perceived need for smartphones in the workplace and how at ease the participants’ felt on using the device in front of patients and ward staff.

## Methods

### The intervention

The iDoc project was established in 2009 when we offered newly qualified doctors in Wales PDAs preloaded with medical textbooks [19]. These textbooks were especially presented for smartphone usage and include a cross-searchable facility. In the second phase we offered our participants a preloaded smartphone [18]. Following feedback from the evaluation, including an expressed reluctance to carry two devices (the iDoc phone and their own, often more up-to-date device), in Phase 3 we provided a licence key which the newly qualified doctor participants used to download the Dr Companion© software and five key texts onto their own device. Once downloaded, use was internet-free. The texts on the iDoc app were: the British National Formulary - BNF, the Oxford Handbook of Clinical Medicine, the Oxford Handbook of Emergency Medicine, the Oxford Handbook of Clinical Specialities and the Oxford Handbook of Clinical Surgery. The expectation was that this supplementary learning tool would enable doctors easily to consult the explicit knowledge provided on the app which would support their clinical practice. Phase 3 of the iDoc project ran for 12-months from August 2012 and evaluation data from this phase is our focus here.

The iDoc project participants are Foundation doctors. The UK Foundation Training Programme bridges the gap between medical school and specialist training. The number of Foundation Year 1 (F1) trainee doctors in Wales in 2012/13 was 322. Participation in the iDoc project was voluntary. Although participants were required to complete a baseline questionnaire in order to access the resource, they were not prevented from using it if they subsequently chose not to take further part in the evaluation.

### Survey design and administration

The baseline questionnaire collected data on the use of workplace information sources, including the use of mobile devices (frequency, type, usefulness and variation in use). The baseline questionnaire was issued when participants were newly in post. Participants completed an exit questionnaire at the end of the data collection phase (July 2013). The exit questionnaire included questions from the baseline, along with additional questions to explore the effects of the intervention. All questions were optional. Questionnaire design was informed by findings from the previous phases (along with focus group discussions during the initial set up of the iDoc intervention programme). Questionnaires were confidential but not anonymous.

Research ethics approval for the iDoc evaluation was obtained from Cardiff University (02/12/2010).

### Data analysis

In this paper we present analysis of the relationship between the data at baseline and exit.

Matched data from baseline and exit questionnaires were entered into SPSS v.20. All variable frequencies were reviewed. As the data were ordinal, a non-parametric statistical test (Wilcoxon Signed Rank test) was used to explore relationships between variables. In line with statistical assumptions, results were considered significant when the *p*-value was less than 0.05 [20].

## Results and discussion

### Survey respondents

Baseline and exit questionnaires were completed by 125 F1 trainees, representing 39% of the total of F1s in Wales at that time. From those who disclosed their gender, 54% of respondents were female (n = 67).

### Use of the iDoc app by junior doctors

Respondents were asked to indicate their use of the iDoc app. Of the sample, 91% (n = 114) reported using the app for more than seven months. Just over half the participants (n = 65) reporting using the app daily (see Table 1).

**Table 1:** **Period and frequency of iDoc app usage (exit questionnaire)**

	*Not used % (n)*	*<1 month % (n)*	*1-6 months % (n)*	*> = 7 months % (n)*	*Total*
For how long have you been using iDoc app?	2% *(3)*	1% *(1)*	6% *(7)*	91% *(114)*	**125**
	***Never % (n)***	***Occasionally % (n)***	***Weekly % (n)***	***Daily % (n)***	***Total***
How often last month did you use iDoc app?	8% *(10)*	8% *(10)*	32% *(40)*	52% *(65)*	**125**

### Information sources used by trainee doctors in the workplace

Respondents were also asked about their use of alternative resources. Data generated by the questionnaires at baseline and exit showed the most frequently used information sources in the workplace *on a daily basis* were: senior medical staff (75% at baseline; 84% at exit); peers (69% baseline; 67% exit); other staff in the medical/nursing team (53% baseline; 58% exit) and the internet (62% baseline; 35% exit). See Table 2.

**Table 2:** **Use of information sources in the workplace**

*Information source*	*Never % (n)*	*Rarely % (n)*	*Monthly % (n)*	*Weekly % (n)*	*Daily % (n)*	*Total*
**Seniors**						
Baseline	0% *(0)*	0% *(0)*	4% *(7)*	20% *(31)*	75% *(87)*	**125**
Exit	1% *(1)*	0% *(0)*	1% *(1)*	14% *(18)*	84% *(105)*	**125**
**Peers**						
Baseline	0% *(0)*	2% *(2)*	6% *(7)*	24% *(30)*	69% *(85)*	**124**
Exit	1% *(1)*	2% *(3)*	4% *(5)*	26% *(32)*	67% *(84)*	**125**
**Other staff**						
Baseline	0% *(0)*	5% *(6)*	9% *(11)*	34% *(42)*	53% *(66)*	**125**
Exit	3% *(4)*	2% *(3)*	3% *(4)*	33% (41)	58% (71)	**123**
**Internet**						
Baseline	1% *(1)*	2% *(3)*	7% *(9)*	28% *(35)*	62% *(77)*	**125**
Exit	2% *(3)*	6% *(8)*	16% *(20)*	40% *(50)*	35% *(44)*	**125**
**Hard-copy text/journals**						
Baseline	2% *(3)*	11% *(14)*	9% *(11)*	38% *(47)*	39% *(49)*	**124**
Exit	17% *(21)*	25% *(31)*	21% *(26)*	29% *(36)*	8% *(10)*	**124**
**Electronic texts PC**						
Baseline	9% *(11)*	27% *(34)*	28% *(35)*	24% *(30)*	11% *(14)*	**124**
Exit	22% *(27)*	30% *(36)*	28% *(34)*	18% *(22)*	2% *(3)*	**122**
**Lecture notes**						
Baseline	20% *(25)*	33% *(41)*	17% *(21)*	26% *(32)*	5% *(6)*	**125**
Exit	62% *(76)*	32% *(39)*	4% *(5)*	2% *(3)*	0% *(0)*	**123**

In terms of using workplace information resources, results displayed some significant changes during the year. Hard-copy textbooks/journals were reported to be accessed daily by only 8% of participants at exit compared to 39% at baseline, a significant decrease in their use during the year (*Z* = −6.326, *p* < 0.001). Likewise, use of electronic textbooks/journals accessed via a PC also declined significantly (*Z* = −3.004, *p* < 0.003). The percentage of respondents who *never* accessed lecture notes increased significantly (20% at baseline; 62% at exit; *Z* = −6.758, *p* < 0.001). Use of the internet as a workplace resource by participants also decreased significantly (*Z* = −4.646, *p* < 0.001) during this period, although it still remained a source of daily information in the workplace for 35% of participants at exit.

In terms of people-based resources, a significant difference was observed in the use of senior medical staff by participants (*Z = −*4.646, *p =* 0.001) where daily use increased from 75% to 84%. No significant difference in the use of peers and other staff as workplace information sources were found.

### Using mobile technology in the workplace

We asked respondents to rate on a scale of 1-10 whether they thought there was a place for smartphone technology in the workplace (where 1 = 'no place' and 10 = 'essential'). Ninety-two per cent (n = 115) of respondents gave a rating of 7 or more in the baseline questionnaire, a proportion that remained consistent at exit (95%; n = 119). However the percentage of respondents rating 10 along the Likert scale, indicating they thought smartphone use had an ‘essential’ place in the workplace, significantly increased from 20% (n = 25) to 37% (n = 46) (*Z* = −4.050, *p* = 0.001).

Questions in the baseline and exit questionnaires asked respondents to indicate whether they would feel comfortable using a mobile device containing textbooks in front of patients and senior medical staff in the workplace (see Table 3). At baseline, 33% (n = 41) of participants strongly agreed or agreed that they would feel comfortable using a mobile device containing textbooks in front of patients. At exit the percentage agreeing or agreeing strongly significantly increased (45%; *Z* = −2.491, *p* = 0.013). For using a smartphone containing textbooks in front of senior medical staff, the exit data showed that 73% of participants strongly agreed or agreed that they would feel comfortable, an increase compared to baseline responses (61%). The most notable, and significant, shift observed in response to this question was for those who *strongly* agreed, 7% at baseline compared to 29% at exit (*Z* = −3.111, *p* = 0.002). These data suggest a growing sense of comfort in using the smartphone app in front of patients and seniors.

**Table 3:** **Comfort using a mobile device containing textbooks in front of patients and seniors**

*Question*	*Strongly agree % (n)*	*Agree % (n)*	*Disagree % (n)*	*Strongly disagree % (n)*	*Total*
**I will feel comfortable using a mobile device containing textbooks in front of patients**					
Baseline	4% *(5)*	29% *(36)*	52% *(65)*	15% *(19)*	**125**
Exit	19% *(24)*	26% *(32)*	39% *(48)*	16% *(20)*	**124**
**I will feel comfortable using a mobile device containing textbooks in front of seniors**					
Baseline	7% *(9)*	54% *(67)*	34% *(42)*	5% *(6)*	**124**
Exit	29% *(36)*	44% *(54)*	21% *(26)*	6% *(8)*	**124**

## Discussion

Participants in the iDoc app intervention were shown to have made use of the resource within workplace settings, which were primarily hospitals within Wales. Most reported having used the iDoc app over the last 12 months, with just over half indicating daily use. The iDoc app usage was associated with a reported decrease in the use of hard-copy textbooks/journals, electronic textbooks/journals via a PC, lecture notes and use of the internet as a workplace information source of choice. In addition to the observed changes in usage pattern, it is perhaps unsurprising that participants felt strongly that there was a place for smartphone technology in the workplace. An understanding of the clinical workplace context where internet connection is unreliable, ward computers are few in number and up-to-date textbooks difficult to locate, helps to explain the positive response to an internet-free mobile resource which provides reliable, up-to-date information and supports knowledge acquisition [7]. By providing the trainees with access to more medical textbooks, more readily, the iDoc app had the effect of supplementing the learning of those taking part in the study and the survey data revealed how app usage replaced traditional hard copy texts and PC-based resources.

Non-significant differences were found between baseline and exit data with regard to using peers and other staff as sources of information. The notable, significant, increase in the use of senior medical staff as sources of information contrasts with reports which express concern about doctors’ dependency and over-reliance on technology [17]. The newly qualified doctors in our sample were more, rather than less, inclined to seek guidance from their seniors. We suggest that mobile technology does not replace human resources and that in our experience, newly qualified doctors as they develop their learning though participation in the workplace [7], are successfully able to navigate between different types of resources, identifying when one is more appropriate than the other. Further, when we consider responses from participants with regard to reported access and usability, it may be that this technology has the potential to create the opportunity to develop deeper learning through what is a period of transition for newly qualified doctors, offering dynamic scaffolding and providing greater learner access to knowledge whilst participating in the workplace [7].

Statistically significant differences indicate that over the course of the year participants in the sample became increasingly more comfortable using their smartphones containing textbooks in the presence of patients and senior medical staff. This finding suggests that continued use of the resource may contribute to overcoming potential barriers to using a mobile phone in the clinical workplace where purpose of use may be unclear. However, although a shifting pattern can be identified, high degrees of discomfort remain. An intervention in medical education and practice which involves mobile technology raises questions of ethics, etiquette and equality of access. Opportunities for innovation need to be encouraged and not just acknowledged [21] and it is clear that more needs to be done to support doctors in their use of available technology. This has implications for training and resources.

Although many participants in the project were highly engaged, the overall uptake amongst newly qualified doctors in Wales was low (although we note that we have reported here only those who completed exit questionnaires). This is surprising, as younger doctors might be considered early adopters of technology; smartphone ownership is ubiquitous in this group. Cost was not an issue as the Wales Deanery provided the app free-of-charge which ran on trainees’ own devices. Low uptake limits the generalizability of our findings. The reasons for this might include the widespread use of alternatives such as UpToDate or numerous other apps, which might lessen the appeal of a textbook app, although one especially designed for mobile use [22]. Reluctance to be involved in a research project during a period of career transition might be another reason for low uptake, and this has substantial implications for innovations in medical education which are accompanied by robust evaluation.

## Conclusion

The transition from medical student to new doctor is a time of intense change in responsibility and practice. The advent of increased responsibility and decision-making can be challenging. Our findings indicate that access to a mobile app enabling timely, internet-free access to key textbooks supports the learning and practice of newly qualified doctors during this critical phase of development. Interestingly, results display an increase in use of senior colleagues by participants’ after the period of iDoc app use. Rather than an over-reliance on information from the app in decision-making, these findings suggest that the app was used strategically to complement, not replace discussion with members of the medical team. Participants’ uncertainty about using a mobile device with textbook app in front of others was shown to ease over time. Further enquiry will be needed to establish whether the smartphone is an essential tool in the doctor’s kit.

## Abbreviations

App: Application software
F1: Foundation year 1
iDoc: Name of the app
PC: Personal computer
PDA: Personal digital assistant
SPSS: Statistical package for the social sciences
UK: United Kingdom
